# Subtractive and differential hybridization molecular analyses of *Ceratitis capitata *XX/XY versus XX embryos to search for male-specific early transcribed genes

**DOI:** 10.1186/1471-2156-15-S2-S5

**Published:** 2014-12-01

**Authors:** Marco Salvemini, Rocco D'Amato, Valeria Petrella, Domenica Ippolito, Giuseppe Ventre, Ying Zhang, Giuseppe Saccone

**Affiliations:** 1 Department of Biology, University of Naples Federico II, 80134 Naples, Italy; 2 Laboratorio Analisi Cliniche "La Salute", 84086, Roccapiemonte, Salerno, Italy

**Keywords:** male-specific, Y chromosome, sexing, embryonic, Suppression Subtractive Hybrydization, Mirror Orientation Selection, Differential Screening Hybridization, SIT

## Abstract

The agricultural pest *Ceratitis capitata*, also known as the Mediterranean fruit fly or Medfly, is a fruit crop pest of very high economic relevance in different continents. The strategy to separate *Ceratitis *males from females (sexing) in mass rearing facilities is a useful step before the sterilization and release of male-only flies in Sterile Insect Technique control programs (SIT). The identification of genes having early embryonic male-specific expression, including Y-linked genes, such as the Maleness factor, could help to design novel and improved methods of sexing in combination with transgenesis, aiming to confer conditional female-specific lethality or female-to-male sexual reversal.

We used a combination of Suppression Subtractive Hybrydization (SSH), Mirror Orientation Selection (MOS) and differential screening hybridization (DSH) techniques to approach the problem of isolating corresponding mRNAs expressed in XX/XY embryos versus XX-only embryos during a narrow developmental window (8-10 hours after egg laying, AEL ). Here we describe a novel strategy we have conceived to obtain relatively large amounts of XX-only embryos staged at 8-10 h AEL and so to extract few micrograms of polyA+ required to apply the complex technical procedure. The combination of these 3 techniques led to the identification of a Y-linked putative gene, *CcGm2*, sharing high sequence identity to a paralogous gene, *CcGm1*, localized either on an autosome or on the X chromosome.

We propose that *CcGm2 *is a first interesting putative Y-linked gene which could play a role in sex determination. The function exterted by this gene should be investigated by novel genetic tools, such as CRISPR-CAS9, which will permit to target only the Y-linked paralogue, avoiding to interfere with the autosomal or X-linked paralogue function.

## Background

The agricultural pest *Ceratitis capitata*, also known as the Mediterranean fruit fly or Medfly, belongs to the Tephritidae family, which includes a large number of other fruit crop damaging pest species. Medfly was the first non-drosophilid genetically transformed fly, paving the way for new biotechnology-based pest control strategies [1,2]. Furthermore, it is an experimentally tractable model, in which transient and transgene-mediated RNAi have also been successfully used [3,4].

The deep genetic knowledge gained in the model organism *Drosophila*, following decades of brilliant basic genetic studies, was an important premise to gain genetic information and know-how to start developing improved methods for pest control. In particular, improved methods for sexing males in *C. capitata *can be useful for the Sterile Insect Technique (SIT) and for the RIDL (Release of Insects carrying Dominant conditional Lethal genes) [5-9]. New genetic knowledge of *C. capitata *sex determination is already being used in the design of novel transgene-based sexing alternatives [4,10,11].

The *C. capitata *Y chromosome promotes male sex determination by a masculinizing factor (M) located on the long arm nearby the centromeric region [12]. The M factor, which is still to be molecularly isolated, exerts its role either directly or indirectly by switching OFF the *transformer *(*Cctra*) splicing regulatory function, required for female sex determination in XX individuals [3]. The *C. capitata *male/female sex determination occurs during the first hours of embryogenesis (5-10 hours) and is maintained by the ON/OFF epigenetic state of the *transformer *gene [13-16]*. Cctra *encodes a serine arginine rich RS protein (RS= rich in serine and arginine), which seems to act as a splicing factor together with CcTRA-2 SR (SR= rich in serine and arginine and containing the RRM RNA binding domain) protein to promote female-specific splicing of *Cctra, Ccdoublesex *(*Ccdsx*) and *Ccfruitless *(*Ccfru*) pre-mRNAs [17]. In female adult XX flies, the *Cctra *transcripts are spliced in the female mode, leading to mRNAs which encode a putative 429 aa-long CcTRA protein. In adult males, *Cctra *produces only longer transcripts containing male-specific exons. The presence of in-frame stop codons interrupts prematurely the putative translation of CcTRA^F ^and the male-specific transcripts encode only short and most likely non-functional CcTRA^M ^peptides ranging between 37 and 99 aa in length [3]. *Cctra *transcripts spliced in the adult female-specific form are also present in unfertilized eggs and in mixed XX/XY embryos collected within the first 4 hours after egg laying, AEL [3; data not shown]. Transcripts spliced in the male-specific form appear at 5-10 hours AEL in XX/XY embryos but not in XX-only embryos (data not shown). Gabrieli *et al*. (2011) presented similar *Cctra *splicing pattern data on sexed individual *C. capitata *embryos [18]. Hence, we expect that the Y-linked *M *factor, either directly or indirectly, is able to switch OFF *Cctra *by promoting its male-specific splicing and that *M *transcripts are transcribed from the Y-linked locus during this time window only in XY embryos. This regulatory pathway has been molecularly compared in *C. capitata *and *Drosophila melanogaster*, which was used as a reference to isolate its key *C. capitata *orthologous regulatory genes [19,20]. Interestingly, *C. capitata *XX fertile adult males are produced following *Cctra *or *Cctransformer-2 *(*Cctra-2*) transient embryonic RNAi, indicating that the Y chromosome contributes to maleness essentially only with the M factor, but not with fertility genes [3,17], in contrast to *Drosophila *Y chromosome which bears six single-copy genes essential for male fertility [21].

The *Drosophila *and *Ceratitis *sex determination cascades diverge upstream of *tra*. In *Drosophila *the female-specific SXL splicing regulator is produced in response to 2 doses of the X-linked elements (XX; X-linked signaling elements, XSE) and promotes female-specific splicing of *tra *whereas in *C. capitata *the Y-linked M switches OFF the otherwise maternally activated *tra *female-specific positive autoregulation [3,17]. However, upstream genes of the *C. capitata *sex determination pathway, such as the Y-linked maleness factor are still elusive [13]. Furthermore, the orthology based approach to the search for sex-specifically expressed genes of *C. capitata *has reached a "dead end" and novel molecular genetic strategies are needed to identify key regulators of sex determination controlling the upper segment of this genetic pathway [13,22].

Differential hybridization patterns of XX versus XY genomic DNA led to the isolation of first *C. capitata *Y-derived DNA clones, which revealed the presence of highly repetitive elements and transposons [23]. More recently, Representational Difference Analysis (RDA) and fluorescence *in-situ *hybridization (FISH) approaches were used to investigate the Y chromosome of another related Tephritidae species, *Bactrocera oleae*, an olive tree fruitfly pest, that led to the isolation of Y-linked genomic sequences related to transposons or importin-like genes which are possibly also transcribed [24]. These studies pointed out that the Tephritidae Y chromosomes are heterocromatic, plenty of transposon-related sequences, of repetitive elements and pseudogenes more often than genes containing full length ORFs.

Subtractive hybridization is an attractive and ingenious method for enriching differentially expressed genes, firstly used more than 5 decades ago, and greatly improved by adding adapters to cDNAs to selectively amplify by PCR the tester cDNA [25] and more recently by introducing the new concept of Suppression Subtractive Hybridization PCR (SSH PCR), in which differentially expressed genes could be normalized and enriched over 1000-fold in a single round of hybridization [26]. The SSH technique is based on the suppression PCR effect that is mediated by long inverted terminal repeats attached to the ends of DNA fragments.

This technique has been used with success to isolate differentially expressed genes for example in different social insect castes [27], in specific insect tissues [28], as well as, in other eukaryotic systems [29-31]. Furthermore SSH led to the identification of *C. capitata *genes expressed in male accessory glands or during embryos cellularization, as well as for example a female-specifically expressed gene in the mosquitoes *Aedes aegypti *[32,33].

We expect that during early stages of *C. capitata *embryogenesis when male/female sex determination takes place 1) the number of genes expressed only in XY embryos from the Y chromosome is relatively low and 2) that one or few of these transcripts is/are derived from the *M *locus, which dictates maleness. We planned to search for male-specific transcripts expressed in *C. capitata *XY embryos at 8-10 hours after egg laying (AEL) using a molecular approach that reduces the number of putative genes needed to be screened for differential expression.

In this study, we performed the Suppressive Selective Hybridization (SSH) molecular subtraction method for a "males/females minus females" between embryonic transcripts and we report the generation of a *C. capitata *embryonic cDNA library enriched male-biased transcripts. We used a mixed XX/XY embryos samples as a tester and XX-only sample as a driver. We improved the selection of real male-specific positive clones by adding two additional steps: 1) the Mirror Oriented Selection (MOS) procedure [34] and 2) differential screening hybridization (DSH; PCR-Select Differential Screening Kit) of the subtracted and filter-spotted cDNA library by radioactive probes [26,35,36].

We obtained a subtracted plasmid library containing 2.5 × 10^4 ^clones. Out of 410 randomly selected clones, 24 of them were identified as putatively male-biased transcripts and 1 was validated by RT-PCR and PCR as a male-specific Y-linked gene.

## Results

### Suppression subtractive hybridization

As a preliminary step for molecular subtraction, we set up, in parallel, two crosses in two large cages (see Methods). In the first cage, 800 XY males crossed with 1600 XX females produced mixed XX/XY embryonic progeny (the tester, which contains transcripts to be enriched); in the second cage, 800 XX "special" males (produced using the transgenic sexing strain Cc 5.3; see Methods and [4]) crossed with 1600 XX females produced female-only XX embryonic progeny, (the driver to be used to subtract, see Methods). We collected embryo samples from the two crosses every 2 hours (during 24 hours non-stop embryos collection), and left them to develop until developmental stage of 8-10 hours AEL. We pooled all the 12 + 12 embryo samples and finally collected about 1 ml of XX/XY and 1 ml XX-only embryos. We extracted high quality total RNA (using cesium chloride density gradient) and we purified by affinity chromatography 2 micrograms of polyA+ mRNAs from each sample.

**Figure 1:**
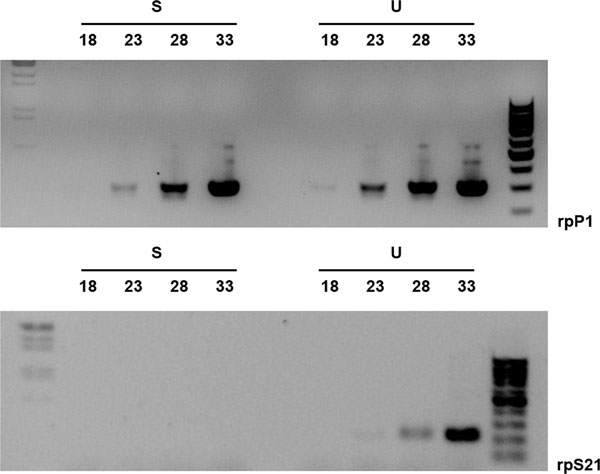
**Analysis of SSH subtraction efficiency**. Gel electrophoresis (upper rpP1, lower rpS1), showing expected PCR products after 18, 23, 28, and 33 cycles of PCR amplification. The quantity of transcripts from the constitutively expressed Ceratitis rpP1 and rpS21 gene was compared between subtracted (S) and unsubtracted (U) cDNA samples. As expected, there is a reduction in the subtracted cDNAs, which is more pronounced for rpS21.

We applied Suppression Subtractive Hybridization (SSH), following the manufacturer's instructions, and produced a forward subtracted library potentially enriched in male-specific/male-biased mRNAs (tester minus driver: XX/XY mixed embryonic cDNAs subtracted with XX-only embryonic cDNAs). We also obtained as a control a reverse subtracted library (driver minus tester: XX-only cDNAs subtracted with the XY/XX cDNAs). We partially validated that the subtraction had indeed taken place by comparing the reduction of constitutive (common) transcripts from two *C. capitata *housekeeping genes, *rpP1 *[37] and *rpS21 *[38,39] by semi-quantitative PCR [40]. *rpP1 cDNA *fragments were detected in the non-subtracted cDNA after 18 PCR cycles of amplification and in the subtracted after 23 cycles (Figure 1). Similarly, *rpS21 *cDNA fragments were detected after 28 cycles in the non-subtracted cDNA while they were completely absent in the subtracted cDNAs sample (Figure 1). This marked reduction of both housekeeping mRNAs in the SSH subtracted cDNA suggested that the SSH has possibly reduced the relative quantity of specific group of transcripts and hopefully also the complexity of the cDNA population in the forward library.

### Mirror orientated selection

**Figure 2:**
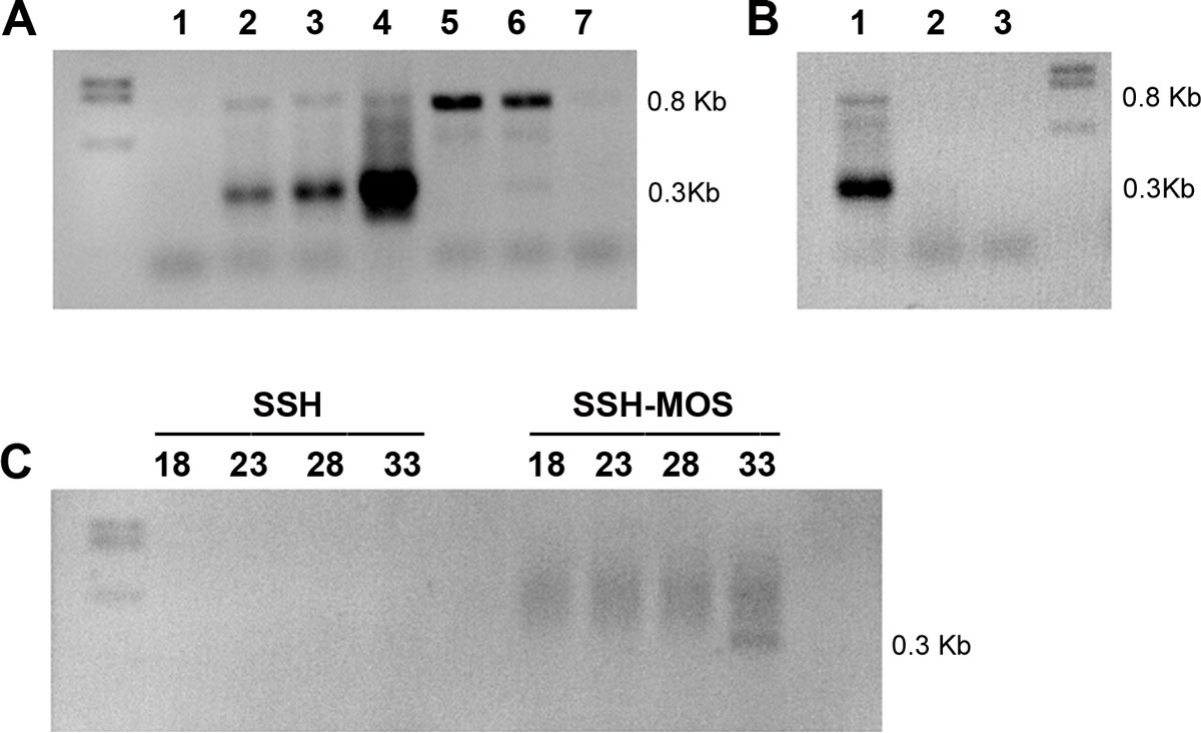
**Analysis of SSH-MOS subtraction efficiency**. A) *Cclap-ps *male-specific transcript expression profile at early embryonic stages: first lane= DNA marker, 1=unfertilized eggs; 2=XX/XY 0-0.5 hour AEL embryos; 3=XX/XY 0.5-3 hours AEL embryos; 4= XX/XY 3-24 hours AEL embryos; 5=XX-only 0-0.5 hour AEL embryos; 6=XX-only 0.5-3 hours AEL embryos; 7= XX-only 3-24 hours AEL embryos. Negative control not shown. B) last lane=DNA marker, *Cclap-ps *transcript expression profile at adult stages: 1=XY adult males; 2=XX adult females; 3=negative control. C) first lane= DNA marker, The subtracted SSH and subtracted SSH-MOS secondary PCR products were amplified using primers for Y-linked *Cclap-ps *transcript. Aliquots of the samples were analyzed by gel electrophoresis after 18, 23, 28, and 33 cycles of PCR amplification.

One of the major drawbacks of the SSH subtraction method is the high number of false positive clones, namely non-differentially expressed (redundant) cDNA species. These undesired background cDNA clones are generated from non-specific annealing of PCR primers or non-ligated adaptors (type-I background) and from redundant cDNA molecules that evade elimination by hybridization (type-II background). In order to reduce the number of background cDNA clones and the complexity of the cDNA mixture we applied the Mirror Oriented Selection (MOS) procedure [34]. MOS utilizes the principle that background molecules have only one orientation of the adaptor sequence, whereas truly differentially expressed molecules have many "progenitors" with adaptor sequences present in both orientations. The result is achieved by removal of one adaptor by restriction digestion, heat denaturation and re-annealing of the resulting cDNA molecules. In this case only hybrid molecules with the adaptors at the opposite ends are exponentially amplified by PCR, while background cDNAs by a linear PCR amplification. We partially validated the MOS procedure, using as reference a very early-transcribed (30' AEL, data not shown) Y-linked *Cclap *pseudo-gene serendipitously discovered in previous studies (*Cclap-ps*, unpub. res.; see Methods) which we expect would be enriched in the forward subtracted library (Figure 2 A-B).

As shown by the RT-PCR analysis at embryonic stages, the *Cclap-ps *0.3 Kb cDNA is amplifiable only in XX/XY embryos, from very early stage of development (30' AEL) but not in unfertilized eggs or XX embryos. The larger cDNA fragment of 0.8 Kb present in both XX/XY and in XX-only embryos is due to non specific amplification. Its cloning and sequencing revealed that it corresponds to a transcript encoding for an highly conserved *Drosophila *protein, unrelated to *Cclap *(Flybase: CG42551; see Methods).

Interestingly, we amplified the *Cclap-ps *cDNA fragment only in the MOS-treated subtracted sample (SSH-MOS) but not in the SSH forward library (Figure 2C). These findings suggested that 1), unexpectedly, the quantity of the male-specific *Cclap-ps *transcript was greatly reduced by SSH in the forward-subtracted library (not amplifiable) but also that 2) MOS background reduction had indeed correctly taken place and that *Cclap-ps *male-specific transcript was enriched in the subtracted SSH-MOS library and detectable again by RT-PCR.

We cloned the cDNAs from forward subtracted SSH-MOS library and plated transformed bacteria, obtaining a plasmid library of 2.5 × 10^4 ^clones.

### Differential screening hybridization

480 forward subtracted plated colonies were randomly picked up and incubated in five 96-well plates (1.2 ml per well - Plate A to E) containing LB medium and ampicillin (Additional file 1 - Figure S1 presents a graphical overview of the differential screening workflow). The bacterial cultures were analyzed individually by PCR amplification for the presence of the plasmid cDNA insert. This led to a total number of 410 subtracted cDNA clones (Additional file 2 - Figure S2).

The PCR products from 410 subtracted cDNA clones were manually blotted on Hybond filter replicates (4 for each plate) (Additional file 1 - Figure S1). Four identical filters were produced for each of the five 96-well plates by arraying spots of each PCR product. The 4 groups of filters, each containing the 5 spotted filters (Plate A-E), were hybridized with either one of the four following complex radioactive-labeled probes: 1) forward-subtracted SSH-MOS tester probe (XY/XX cDNAs minus XX cDNAs), which identifies differentially expressed clones plus false positives. 2) reverse-subtracted SSH tester probe (XX cDNAs minus XY/XX cDNAs) which identifies only false positives as common signals to the first probe, 3) unsubtracted tester probe (XY/XX cDNAs) which identifies differentially expressed clones plus false positives both highly expressed, 4) unsubtracted-driver probe (XX-only cDNAs), which identifies only false positives highly expressed.

**Figure 3:**
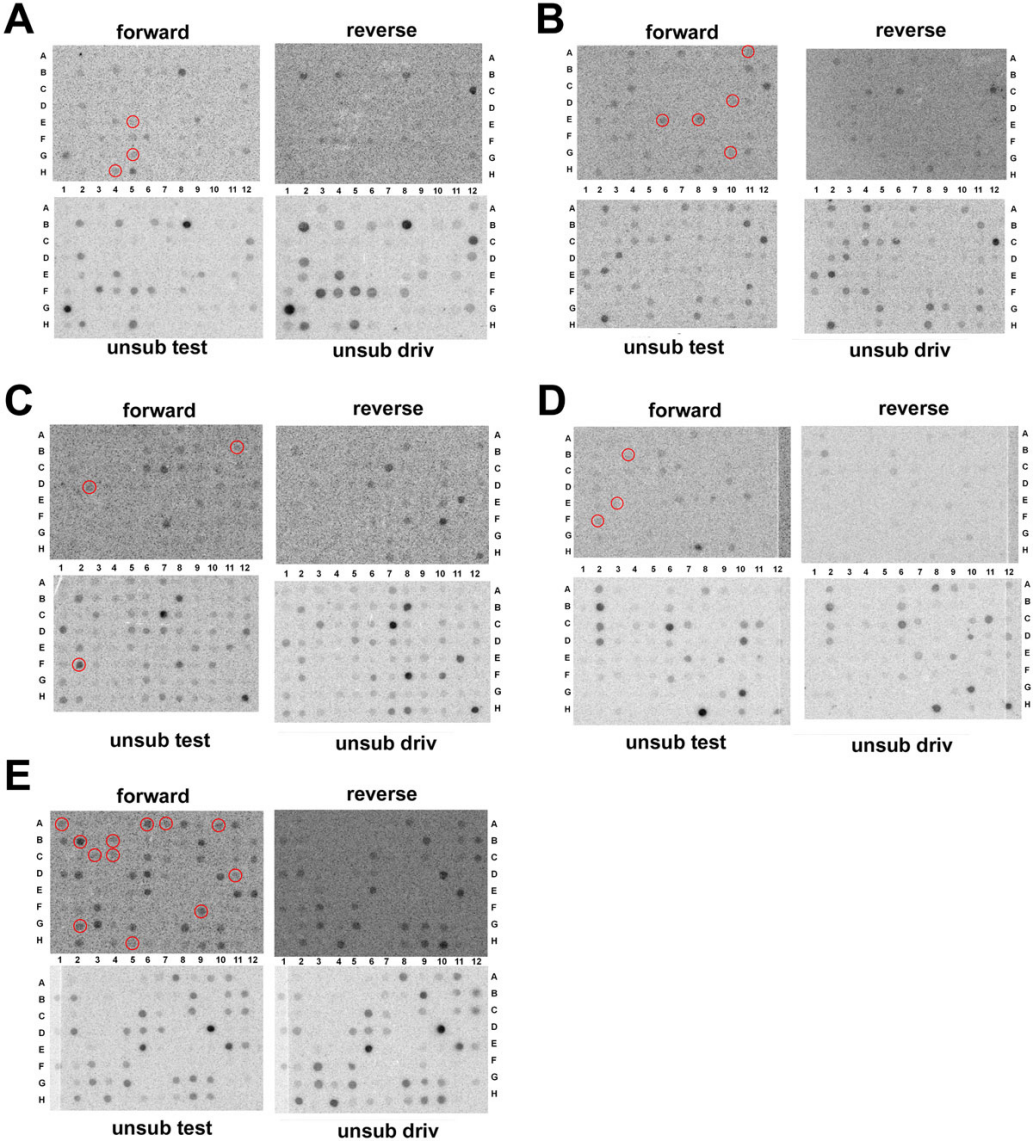
**Differential hybridization screening**. Differentially expressed clones in a 8-10 h AEL embryonic suppression subtraction hybridization (SSH) / mirror orientation selection (MOS) library. 410 randomly selected clones were spotted onto 5 nylon membranes in four replicates (A to E) and hybridized with labelled probe from: subtracted XX/XY vs XX-only SSH/MOS cDNAs (forward); subtracted XX-only vs XX/XY SSH/MOS cDNAs (reverse); unsubtracted XX/XY cDNAs (unsub test); unsubtracted XX-only cDNAs (unsub driv). Hybridization signals were scored visually. Red circles indicate the selected 26 clones.

The clones that hybridize with the forward-subtracted SSH-MOS (XX/XY) and unsubtracted tester probes (XX/XY), but not with the reverse-subtracted SSH (XX minus XX/XY) or unsubtracted driver probes (XX), usually correspond to differentially expressed genes, namely real positive clones (see also the manual of the manufacturer; Methods). Furthermore, those clones from SSH-MOS having no detectable hybridization signals with any probe could still represent differentially expressed transcripts as well as false positives but having a very low abundance. Finally, those clones hybridizing equally with both subtracted probes and unsubtracted probes, are usually the most highly expressed clones, including either real positive or false positive clones. The results of the differential screening are shown in Figure 3 and hybridization signals were scored visually. In our experiment, from 410 clones, 25 of them were identified as hybridizing with the forward-subtracted SSH-MOS tester probe (XX/XY minus XX), but not or only weakly hybridizing with the reverse-subtracted SSH probe. An additional positive clone was identified as hybridizing with unsubtracted tester probe (XX/XY). The 26 clones either failed to hybridize or weakly hybridized with the reverse-subtracted (XX minus XX/XY) and driver (XX) probes. The 26 cDNA sequences when aligned via Macaw software to search for identity/similarity revealed that AE5 and BE6 clones are identical in sequence and length respectively to DB4 and BE8 clones. The 24 different cDNA clones (ranging approximately from 300 to 900 bp) were analyzed by BLASTX revealing that most of them (18 cDNAs) encode for putative ORFs with significant homology with known dipteran proteins (Additional file 3 - Table S1). 6 cDNA clones seem to encode either unknown or weakly conserved proteins or they could correspond to untranslated regions of transcripts.

One experiment of quantitative real time PCR analysis performed without replicates (data not shown) was apparently consistent with male-biased expression of 24 cDNA clones but not with male-specificity for any of the cDNAs. As we were focused on the search of male-specific genes we further analyzed only one clone (BA11) showing the strongest putative male-bias.

### Molecular analysis of BA11 clone

**Figure 4:**
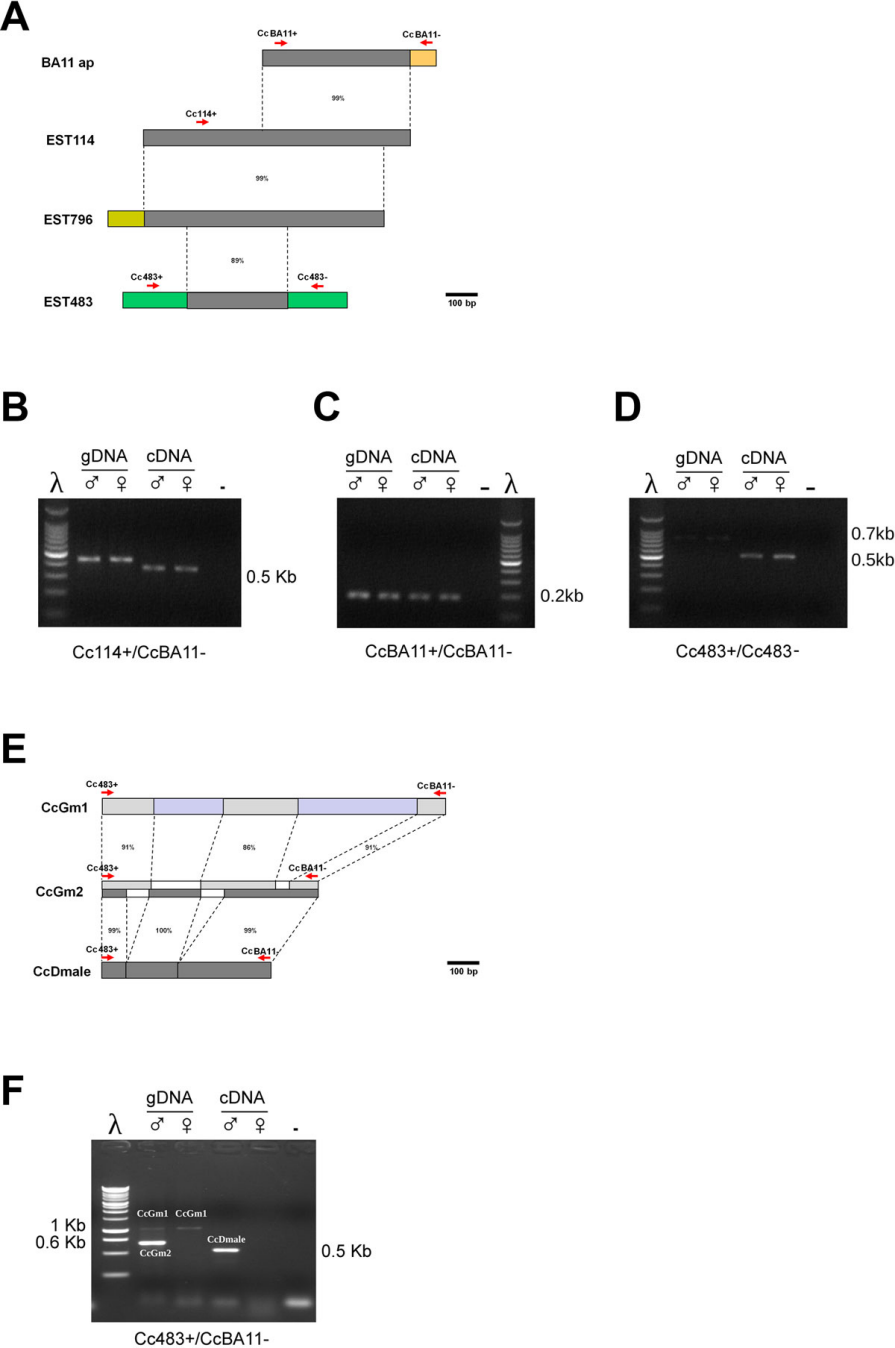
**Molecular analysis of the BA11 clone**. A) Schematic representation of alignments between the BA11 clone and the three corresponding Medfly ESTs. Percentages of nucleotide identities are reported. Red arrows indicate the position of the primers utilized. B) Genomic PCR and RT-PCR product analyses on adult male and female samples with Cc114+/BA11- primers pair. C) Genomic PCR and RT-PCR analyses on adult male and female samples with CcBA11+/CcBA11- primers pair. D) Genomic PCR and RT-PCR analyses on adult male and female samples with Cc483+/483-1- primer pair. E) Schematic representation of alignments between the PCR fragments amplified with Cc483+/CcBA11- primer pair. In light grey alignments between *CcGen1* and *CcGen2* clones and in dark grey alignments between *CcGen2 *and *CcDmale *clones. Percentages of nucleotide identities are also reported. Red arrows indicate the position of the primers utilized. F) Genomic PCR and RT-PCR analyses on adult male and female samples with Cc483+/CcBA11- primer pair, which led to identify a Y-linked paralog.

We used BA11 as an *in silico *probe for BLASTN analyses at NCBI of the *C. capitata *Expressed Sequence Tags (ESTs) database and identified three partially overlapping ESTs (named EST114, EST796 and EST483, ranging from 800 to 700 bp long) which showed respectively identities of 99% over 461 nt, 99% over 378 nt and 95% over 72 bp (see Figure 4A). EST114 and EST796 are derived respectively from embryos and from adult head [41]; they have only four nt differences over 752 bp and correspond most likely to transcripts derived from the same gene (with either SNPs or few DNA sequencing errors).

The EST483 (EST from embryos) compared by BLASTX with the other two ESTs revealed a 470 bp long region showing 80-81% identity, hence it corresponds to a transcript derived from a paralogous gene. BLASTX analysis of the 3 ESTS revealed, as shown previously by the shorter BA11 sequence, sequence similarity with the *Drosophila *CG10803 (isoform B protein) containing the putative RNA binding TROVE domain (Telomerase, Ro and Vault domain).

We asked if any of these sequences are derived from Y-linked genes. The database of Gomulski et al., 2008 was very useful to extend the sequence BA11 information and to design novel primers [41]. So we designed 5 primers on the basis of the BA11, EST114/796, and EST483 sequences (CcBA11+, CcBA11-, Cc114+, Cc483+ and Cc483-; see Figure 4A). A pair of primers specific for the BA11 (CcBA11+, CcBA11-) and the Cc114+/BA11- pair amplified genomic fragments in both sexes (Figure 4B-C). The two EST483 specific primers (Cc483+ and Cc483-) amplified the same fragments in both sexes from genomic DNA (Figure 4D). Interestingly, the combination Cc483+/CcBA11- amplified from genomic DNA a strong male-specific 0.6 Kb long product (*CcGm2*) as well as a very weak 1 Kb long DNA product in both sexes (Figure 4F).

The first 3 combinations of primers, when used in RT-PCR analyses on RNA extracted from adult flies, amplified fragments of comparable length in both sexes (Figure 4B-D). The pair Cc483+/CcBA11- amplified again a male-specific cDNA product (*CcDmale*), which was slightly smaller then the genomic *CcGm2 *(Figure 4E-F). The 3 products were cloned and sequenced, showing that they correspond as expected to two paralogous putative genes, with one most likely Y-linked.

The non sex-specific *CcGm1 *genomic clone contains 4 exonic regions identical to the EST483 cDNA clone and 3 intervening short sequences corresponding to intronic regions (data not shown). The male-specific *CcGm2 *genomic clone contains 3 exonic regions identical to EST114/796 cDNA clones and also 2 intervening introns. *CcGm1 *and *CcGm2 *genomic clones showed an overall 72% DNA sequence identity, confirming that they are most likely paralogous transcribed regions. A BLASTN analysis of a *C. capitata* embryonic mixed sex XX/XY transcriptome led to identity a 1.7 Kb partial transcript containing a 466 aa long ORF but most likely missing 3' end coding and UTR regions (data not shown; Salvemini *et al.*, submitted). The encoded putative protein showed by FlyBlastx analysis an overal 31% aa identity and 46.5% similarity to the *Drosophila *CG10803 encoded protein, as previously observed for the BA11 shorter cDNA clone.

The novel male-specific cDNA *CcDmale *clone obtained by RT-PCR has 100% identity to sequences of the Y-linked *CcGm2 *genomic sequence (and BA11) and only 56% similar to non sex-specific *CcGm1 *genomic sequence. An ORF finder analysis of *CcDmale *clone showed the presence of a 45 aa long proteic sequence having putative Met and Stop codons, showing 42% (19/45 aa) similarity with the *Drosophila *CG10803 TROVE-containing RNA-binding protein.

These findings strongly suggest that *CcGm2 *is a Y-linked putative gene encoding for a shortened protein paralogous to the one encoded by the *CcGm1 *locus. *CcGm1 *gene is either an autosomal or an X-linked paralogue of *CcGm2*. Both genes are transcriptionally active during embryogenesis as well as at adult stages (data not shown).

## Conclusions

The embryonic stage (8-10 hours) used for our molecular subtraction corresponds to a period in which male sex determination has occurred a few hours before, a critical event for sexual development. We have chosen a narrow temporal window of 2 hours to investigate because we expected to find less transcriptional complexity compared with larger embryonic developmental windows. It is well known that complex dynamic of differential transcription underlies the embryogenesis of the model system *Drosophila*. Hence, it was necessary to collect large amounts of embryos from this narrow stage to prepare enough quantity of polyA+ to apply the SSH-MOS-DHS combined technique. So we conceived a strategy based on the fertility of XX males and on their use as fathers of XX-female only progeny.

We expected to identify few male-specific genes expressed so early during embryogenesis and hopefully good Y-linked maleness factor candidates. Only 1 male-specific cDNA clone was obtained by SSH-MOS-DHS approach in a sample of 410 cDNA clones, which were randomly selected from a forward subtracted plasmid library of 2.5 × 10^4 ^cDNA clones. Hence, the overall efficiency of SSH-MOS in isolating male-specifically expressed cDNA clones was 0.002% (1/410). We expect that the subtracted library would contain at least other 60 putative male-specific cDNAs, some of which could eventually correspond to novel Y-linked genes. The simplest way to proceed and get more information on Y-linked genes, would be to sequence by next generation sequencing the entire SSH-MOS subtracted library and use the cDNA sequences in combination with the chromosome quotient (CQ) analysis, a novel approach to systematically discover Y chromosome linked genes [42]. It is interesting to note that also Hall et al., [42] in *Anopheles stephensi *and *An. gambiae*, Gabrieli et al., [24] in *Bactrocera oleae *and Carvalho et al., [21] in *Drosophila melanogaster *had identified very few Y-linked genes using very different methods.

The subtracted male-specific cDNA clone corresponds to a Y-linked putative gene, *CcGm2*, sharing high sequence similarity with a paralogue, *CcGm1*, most likely localized either on autosome or on the × chromosome. Very interestingly the autosomal paralogue encodes a long putative protein, showing significant similarity to a *Drosophila *putative RNA binding protein having the TROVE domain. The *CcGm2 *Y-linked gene however encodes for a short, truncated version of the autosomal (or X-linked) paralogous protein, which could have a different function. Considering that *C. capitata *sex determination is based on sex-specific alternative splicing of *Cctra*, the possibility that the *CcGm1 *Y-linked gene could be involved in controlling splicing is quite attractive although presently very speculative.

A preliminary attempt to functionally investigate *CcGm2 *Y-linked gene by transient embryonic RNAi experiments failed to lead to consistent conclusions because of a frequent non-sex-specific lethality and a subtle not convincing male-specific lethality (data not show). As dsRNA molecules would target both paralogous genes it would be impossible to discriminate between the functions performed by the two paralogues. We propose to use in future experiments the CRISPR-Cas9 mutagenesis method, which permits to target short DNA sequence of 20 bp and discriminate between highly similar paralogous genes, as in our specific case [43-46]. This will require the establishment of this very novel technique also in the Medfly.

## Methods

### Fly strains and crosses

*C. capitata Benakeion *strain flies were reared in standard laboratory conditions at 25°C, 70% relative humidity and 12:12 h light-dark regimen. 800 XX or XY males were mated with 1600 XX females and maintained in large cages (60cm** × **60cm** × **70cm). After 3-4 days, eggs were collected in water dishes for 2 hours and left to develop until developmental stage of 8-10 hours AEL.

We used a *C. capitata* transgenic line inducing masculinisation by a maternal *in vivo *RNAi targeting of *Cctra *mRNAs present in the eggs and early embryos to produce a large number of XX only males, which were crossed with normal XX females to obtain female-only progeny ([4]; Saccone *et al*., manuscript in prep). The cross of XX transgenic females with XX males (either transgenic or not) leads to XX male-only progeny. These males can be used again in crosses with XX transgenic females to produce XX male only progeny or with XX non transgenic females (wild type) to produce XX female only progeny (Saccone et al., manuscript in prep).

### RNA isolation

The total RNA was extracted from 1 ml of collected embryos for each sample, using the standard guanidinium isothiocyanate procedure. The concentration and purity of the RNAs were determined by measuring the absorbance at 260 and 280 nm, and the integrity of the RNA was assessed using denaturing agarose gel electrophoresis. The poly A+ RNAs were isolated using the Oligotex mRNA Mini Kit (QIAGEN) following the manufacturer's instruction.

### Generation of cDNA libraries using SSH and MOS

polyA+ RNA (2.2 μg) from the XX/XY and the XX-only embryonic samples was used to generate cDNA libraries by SSH using the PCR select DNA subtraction kit (CLONTECH Laboratories, Inc.). Two subtractions were performed: forward SSH (subtraction of XX/XY sample from XX-only sample) and reverse SSH (subtraction of XX-only sample from XX/XY sample). Mirror orientation selection (MOS), a modification to eliminate false positive clones, was used as described by Rebrikov et al. (2000) [34]. PCR products from SSH-MOS were cloned into plasmid pGEM-T Easy Vector (Promega) and transformed in *E.coli *cells (Promega). We confirmed the validity of the MOS procedure using an early embryonic male-specific transcript of *C. capitata *as positive control. We isolated this transcript, named *Cclap-ps*, by analyzing the putative ORFs contained in the Y-linked repetitive element 5Kb and performing BLASTN analysis on CcESTs databases (Accession Number: AF115330.1 - 5,642 bp long; [23]). We identified a putative ORF (from position 2764 to 3165 of the 5Kb element) with 56% similarity (protein level) with the *Drosophila *CG13340 gene located on chromosome 2R, which encodes a leucyl aminopeptidase (Lap). The *C. capitata lap *(*Cclap*) ORF is interrupted in its genomic sequence by multiple stop codons, suggesting that it could correspond to a pseudo-gene but on the other hand it could be possible that this genomic region corresponds to an intronic region (data not shown). We designed two specific primers to the most conserved regions of the BLASTX alignment with the *Drosophila* genome (named Y2+ and Y2-) to amplify the Y-linked gene/pseudo-gene in RT-PCR experiments on total RNA extracted adult male and female flies, as well as, from early embryonic samples (see Figure 2A-B). We amplified a prominent band of expected size (0.3 kb) and two low abundance bands of 0.6 kb and 0.8 kb in XY embryos and in adult XY males. The cloning and the sequencing of the 0.3 kb amplification product confirm us that it is the expected 5Kb derived product. This amplification signal is absent in XX-only embryos and in adult females, indicating that it is a male-specific and Y-linked derived transcript. In XX-only embryos, a strong 0.8 kb band was amplified at 30 min and 3 h AEL. The cloning and the sequencing of this cDNA product revealed that it is not derived from *Cclap-ps *and hence has originated by non-specific primer annealing. No amplification was observed in unfertilized X0 eggs.

### Differential hybridization screening of the subtracted libraries

Screening of the subtracted libraries was performed using the PCR-Select Differential Screening Kit (CLONTECH Laboratories, Inc.). Briefly, 480 cDNA clones were amplified by PCR (a sample was run on agarose-gel), NaOH-denatured, blotted on Hybond-N nylon membranes (Amersham Pharmacia Biotech), and UV cross-linked. Five filters with 96 clones each were produced in four replicates. Each of the five filters was screened by differential hybridization with the ^32^P-labeled forward subtracted cDNAs and the ^32^P-labeled tester cDNAs as positive and with the ^32^P-labeled reverse subtracted cDNA and the ^32^P-labeled driver cDNA as negative. Subtracted forward and reverse cDNAs and unsubtracted tester and driver cDNAs were digested with *Xma*I to remove the adaptors, purified using NucleoSpin^® ^Extraction Kit (CLONTECH Laboratories, Inc.), and ^32^P-labeled by random priming (PCR-Select Differential Screening Kit, CLONTECH Laboratories, Inc.). Unincorporated radionucleotides were removed using CHROMA SPIN™-20 Columns (CLONTECH Laboratories, Inc.). Membranes were hybridized overnight at 72°C in ExpressHyb hybridization solution and specific blocking solution (CLONTECH Laboratories, Inc.). Membranes were washed as recommended at 68°C (four times for 20 min each time in 2X standard saline-citrate/0.5% sodium dodecyl sulfate and twice for 20 min each time in 0.2X standard saline-citrate/0.5% sodium dodecyl sulfate) and exposed to Kodak X-OMAT AR film (Eastman Kodak Co.). Plasmid DNA from positive clones was isolated using the Wizard^® ^Plus SV Minipreps DNA Purification System (Promega). The DNA of each sample is sequenced with the Big Dye^® ^Terminator v1.1 sequencing Kit (Applied Biosystem) using the primer T7 and SP6 (0.8 pmol/μl) and then analyzed by BLASTX.

### RT-PCR

RT-PCRs were performed using the Advantage^® ^RT-for-PCR Kit (Clontech) following the manufacturer's instructions. The rpP1(ribosomal protein P1) primer pairs and the rpS21 primer pairs were used as positive control (RpP1+: 5'-TTGCGTTTACGTTGCTCTCG-3'; RpP1-: 5'-AATCGAAGAGACCGAAACCC-3'; RpS21+: 5'- GGTGAATCTGTTGATTTTGTAC-3'; RpS21-: 5'-GCCTTGGTCATCAAACCATC-3'). The RT-PCR expression analysis on *Cclap-ps *transcripts was performed with the Y2 primer pairs (Y2+: 5'-AAGGACTTGTGATTGGATTG-3'; Y2-: 5'-ATGCCGTCGTCCAACATC-3'). The RT-PCR analysis of the BA11 clone was performed with the following primers (Cc114+: 5'-TTAGGACATTTGCCATGGAAT-3'; Cc483+: 5'-CCAGCAGTCGTTCGGTAATAA-3'; Cc483-: 5'-TGTATCGGAATAACGCATCG-3'; CcBA11+: 5'-CGTGGTAATCCTGAAAACAGC-3'; CcBA11-: 5'-CTTACGATCTTCCATGCTTCAC-3').

## Competing interests

The authors declare that they have no competing interests.

## Authors' contributions

GS and MS designed the experiments. GS conceived the project, which is based mainly on the use of the *C. capitata *transgenic strain Cc5.3 to design novel crosses aimed to obtain large amounts of either XX-only females or XX-only males and to apply SSH on XX/XY minus XX embryonic mRNAs. MS improved the crossing schemes to facilitate the maintenance of the transgenic strain and proposed the use of MOS and differential hybridization to improve SSH. MS, GV and GS collected embryos. MS performed SSH-MOS and differential hybridization. GV, RD, VP. DI and YZ maintained strains, performed PCR, RT-PCR, cloning and sequencing. MS, GS and RD performed bioinformatics analysis. GS and MS wrote the paper with inputs from RD and YZ.

## Supplementary Material

Additional file 1 **Figure S1**. Graphic overview of the workflow to produce replica filters for the differential screening analysis. A) PCR selection of clones. B) Replica filter production. Click here for file

Additional file 2 **Figure S2**. PCR selection of clones for the differential screening. In red empty plasmids. In green plasmid with multiple inserts cloned. Click here for file

Additional file 3 **Table S1**. BLASTX analysis results of the 24 SSH-MOS clones selected by differential hybridization screening. Click here for file
