# Supplementary material for: Subtractive and differential hybridization molecular analyses of Ceratitis capitata XX/XY versus XX embryos to search for male-specific early transcribed genes
Source: BMC Genet. 2014 Dec 1;15(Suppl 2):S5. doi: 10.1186/1471-2156-15-S2-S5 (PMC4255797; doi:10.1186/1471-2156-15-S2-S5)

Additional file 1 – Figure S1

Graphic overview of the workflow to produce replica filter for the differential screening analysis. A) PCR selection of clones. B) Replica filter production.

A

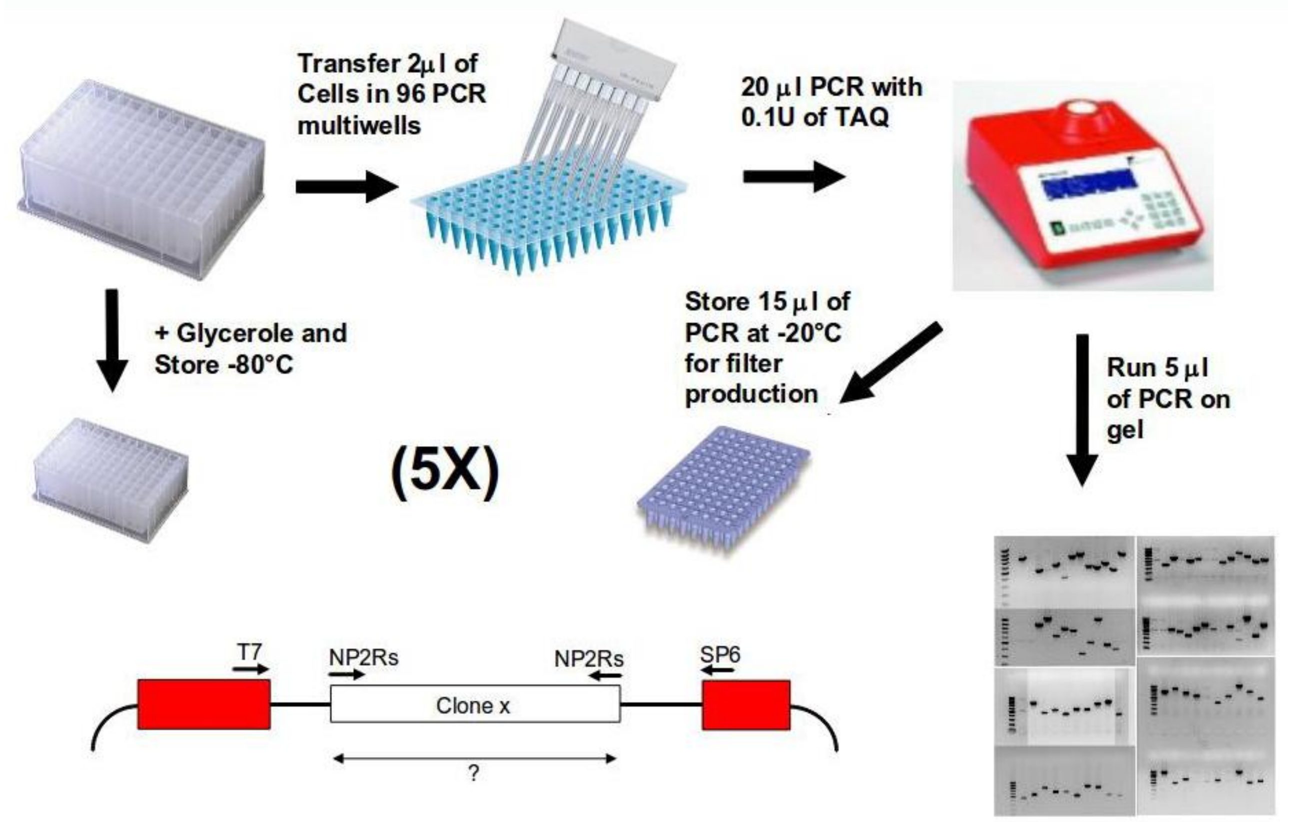

B

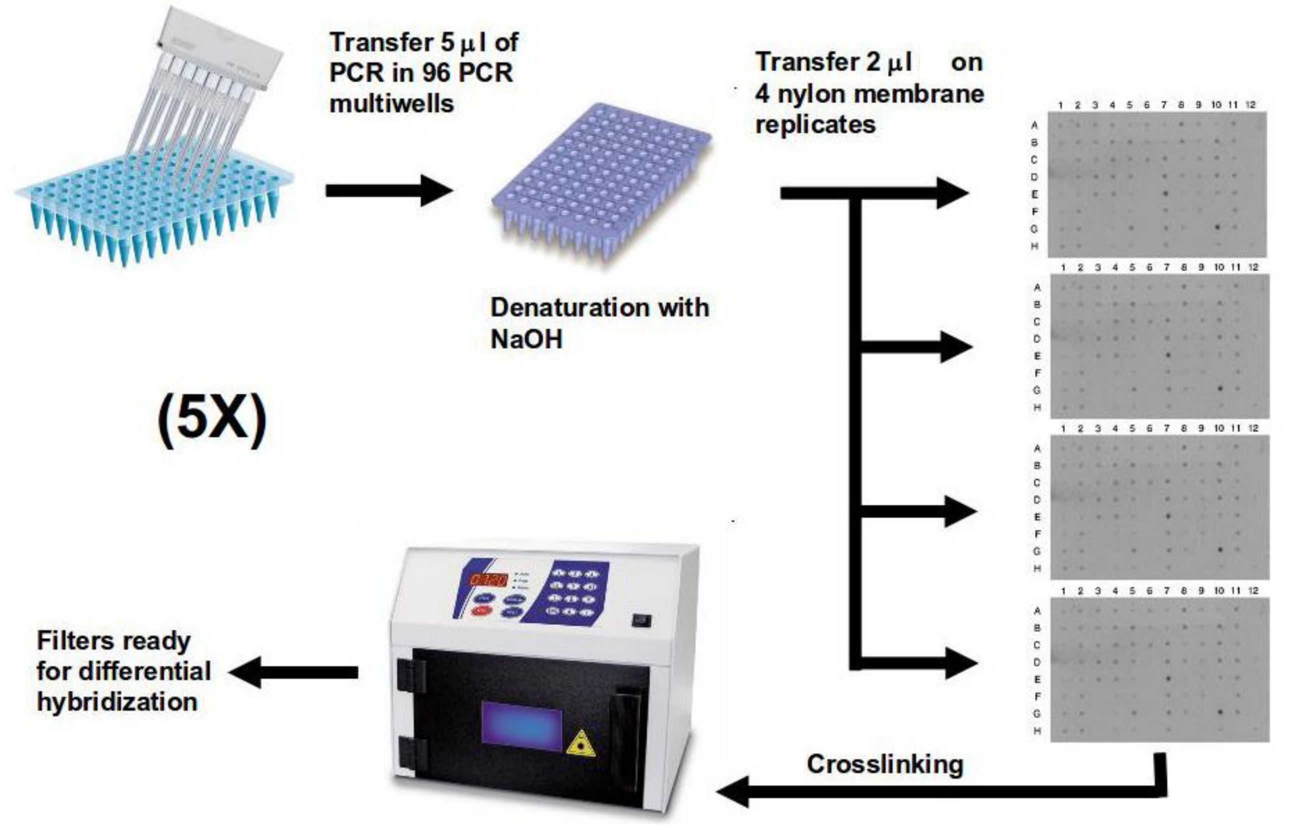

Supplement: Additional file 1 — Figure S1. Graphic overview of the workflow to produce replica filters for the differential screening analysis. A) PCR selection of clones. B) Replica filter production. [file 1471-2156-15-S2-S5-S1.pdf]
