# Supplementary material for: Subtractive and differential hybridization molecular analyses of Ceratitis capitata XX/XY versus XX embryos to search for male-specific early transcribed genes
Source: BMC Genet. 2014 Dec 1;15(Suppl 2):S5. doi: 10.1186/1471-2156-15-S2-S5 (PMC4255797; doi:10.1186/1471-2156-15-S2-S5)
Supplement: Additional file 2 — Figure S2. PCR selection of clones for the differential screening. In red empty plasmids. In green plasmid with multiple inserts cloned. [file 1471-2156-15-S2-S5-S2.pdf]

Additional file 2 – Figure S2

PCR selection of clones for the differential screening. In red empty plasmids. In green plasmid with multiple inserts cloned.

Plate A

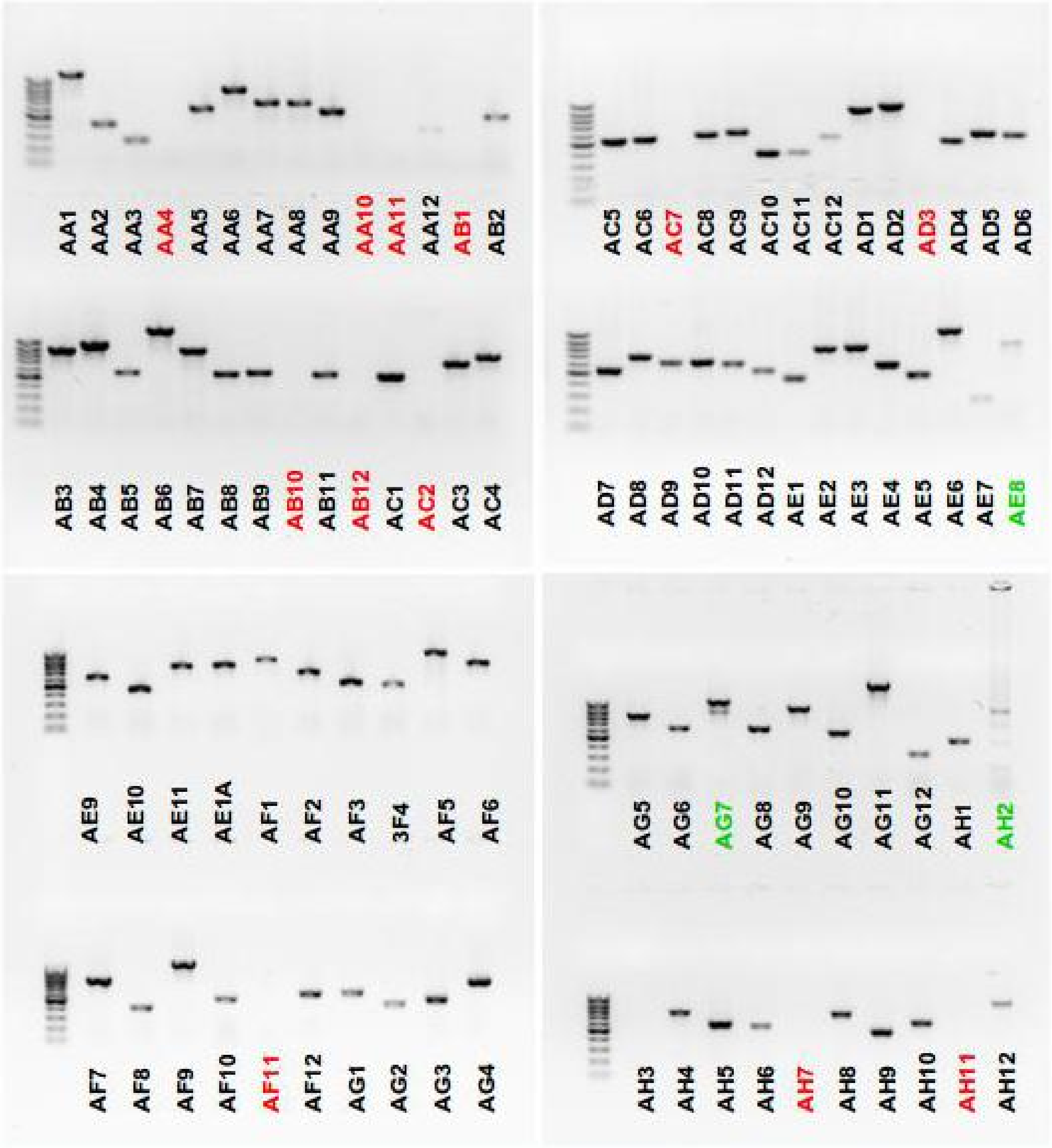

Plate B

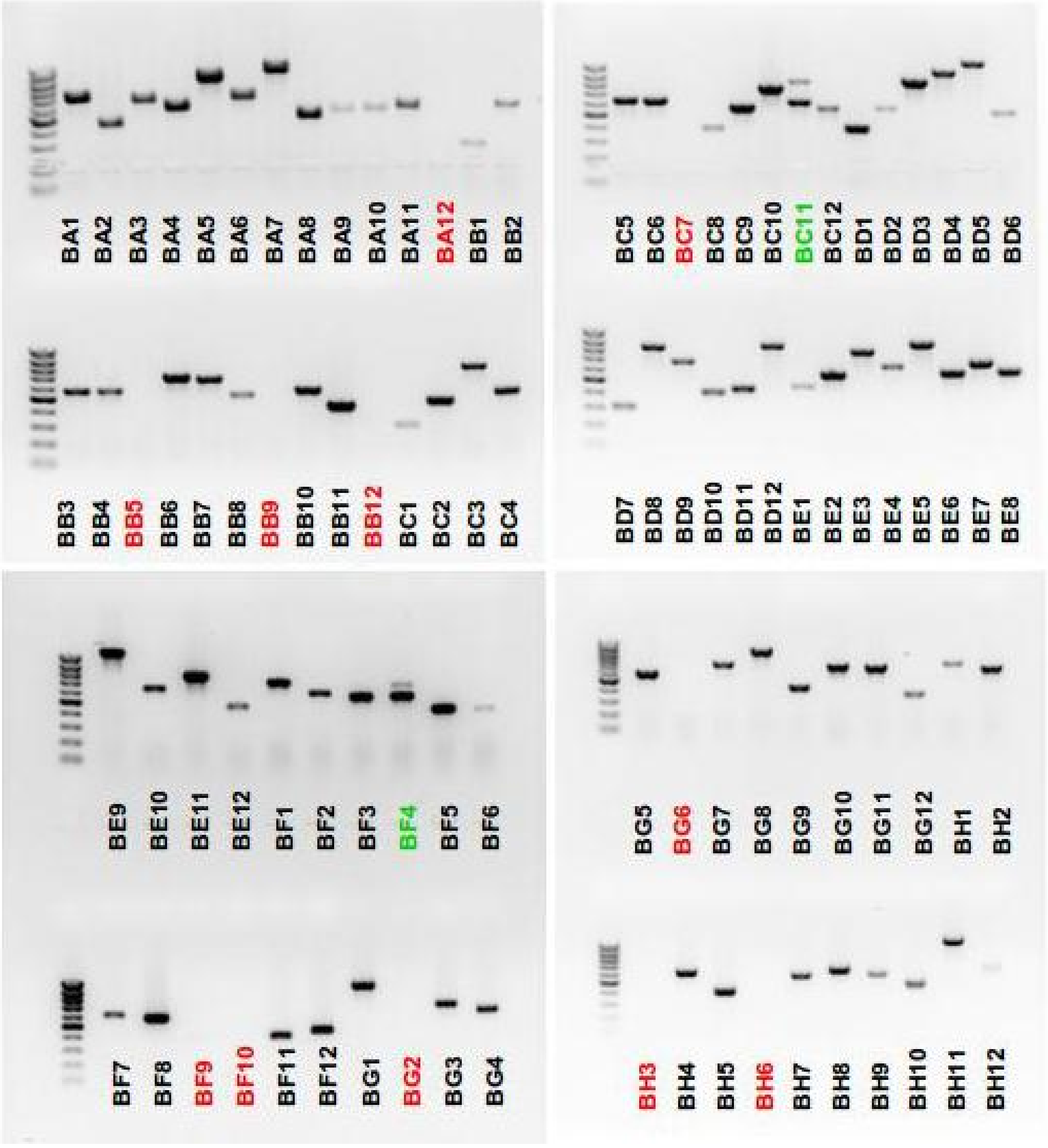
BA1 BA2 BA3 BA4 BA5 BA6 BA7 BA8 BA9 BA10 BA11 BA12 BB1 BB2
BB3 BB4 BB5 BB6 BB7 BB8 BB9 BB10 BB11 BB12 BC1 BC2 BC3 BC4
BC5 BC6 BC7 BC8 BC9 BC10 BC11 BC12 BD1 BD2 BD3 BD4 BD5 BD6
BD7 BD8 BD9 BD10 BD11 BD12 BE1 BE2 BE3 BE4 BE5 BE6 BE7 BE8
BE9 BE10 BE11 BE12 BF1 BF2 BF3 BF4 BF5 BF6
BF7 BF8 BF9 BF10 BF11 BF12 BG1 BG2 BG3 BG4
BG5 BG6 BG7 BG8 BG9 BG10 BG11 BG12 BH1 BH2
BH3 BH4 BH5 BH6 BH7 BH8 BH9 BH10 BH11 BH12

Plate C

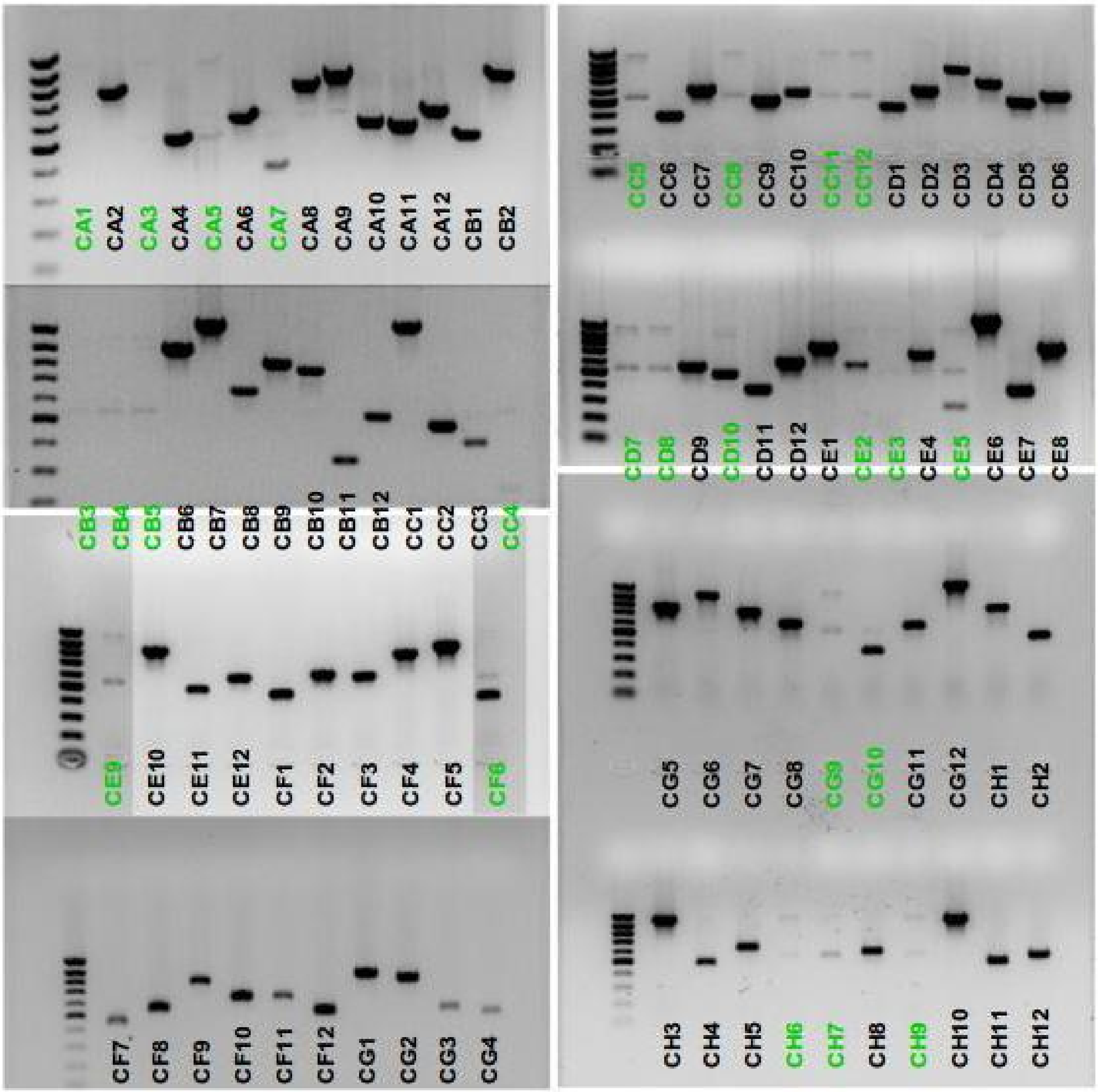
CA1 CA2 CA3 CA4 CA5 CA6 CA7 CA8 CA9 CA10 CA11 CA12 CB1 CB2
CB3 CB4 CB5 CB6 CB7 CB8 CB9 CB10 CB11 CB12 CC1 CC2 CC3 CC4
CE9 CE10 CE11 CE12 CF1 CF2 CF3 CF4 CF5 CF6
CF7 CF8 CF9 CF10 CF11 CF12 CG1 CG2 CG3 CG4
CC5 CC6 CC7 CC8 CC9 CC10 CC11 CC12 CD1 CD2 CD3 CD4 CD5 CD6
CD7 CD8 CD9 CD10 CD11 CD12 CE1 CE2 CE3 CE4 CE5 CE6 CE7 CE8
CG5 CG6 CG7 CG8 CG9 CG10 CG11 CG12 CH1 CH2
CH3 CH4 CH5 CH6 CH7 CH8 CH9 CH10 CH11 CH12

Plate D

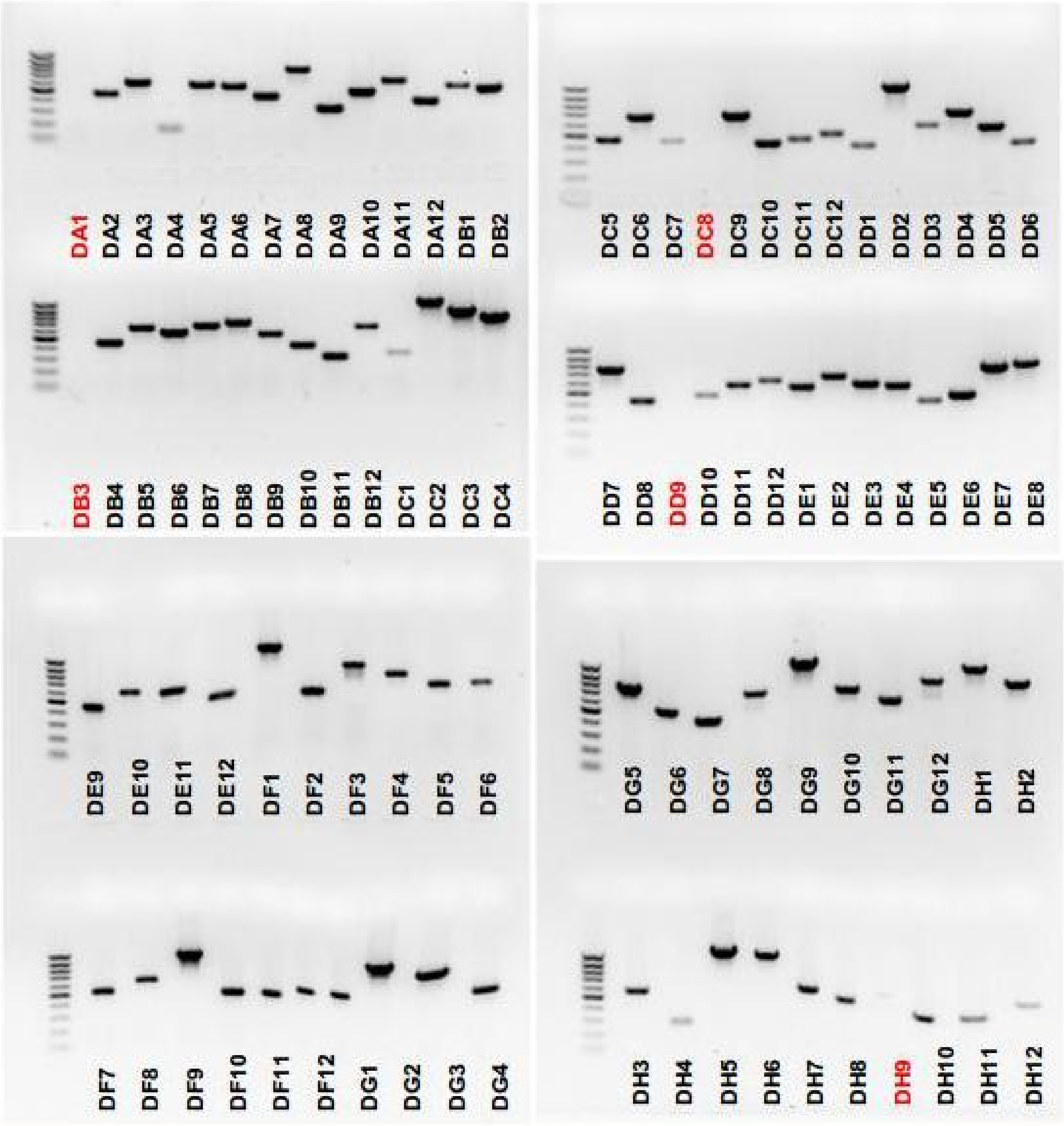

DA1 DA2 DA3 DA4 DA5 DA6 DA7 DA8 DA9 DA10 DA11 DA12 DB1 DB2
DB3 DB4 DB5 DB6 DB7 DB8 DB9 DB10 DB11 DB12 DC1 DC2 DC3 DC4
DC5 DC6 DC7 DC8 DC9 DC10 DC11 DC12 DD1 DD2 DD3 DD4 DD5 DD6
DD7 DD8 DD9 DD10 DD11 DD12 DE1 DE2 DE3 DE4 DE5 DE6 DE7 DE8
DE9 DE10 DE11 DE12 DF1 DF2 DF3 DF4 DF5 DF6
DF7 DF8 DF9 DF10 DF11 DF12 DG1 DG2 DG3 DG4
DG5 DG6 DG7 DG8 DG9 DG10 DG11 DG12 DH1 DH2
DH3 DH4 DH5 DH6 DH7 DH8 DH9 DH10 DH11 DH12

Plate E

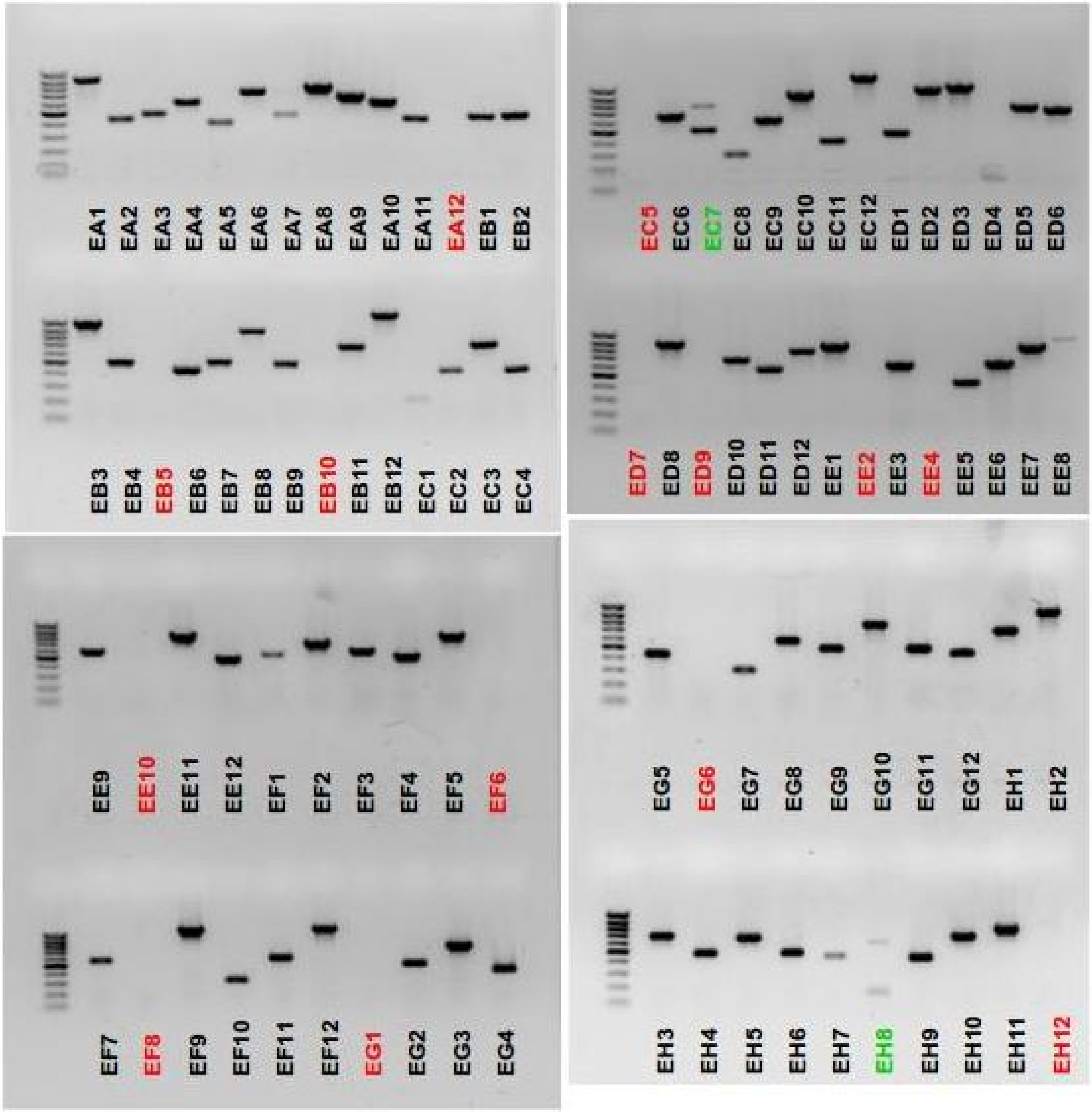

EA1 EA2 EA3 EA4 EA5 EA6 EA7 EA8 EA9 EA10 EA11 EA12 EB1 EB2
EB3 EB4 EB5 EB6 EB7 EB8 EB9 EB10 EB11 EB12 EC1 EC2 EC3 EC4
EC5 EC6 EC7 EC8 EC9 EC10 EC11 EC12 ED1 ED2 ED3 ED4 ED5 ED6
ED7 ED8 ED9 ED10 ED11 ED12 EE1 EE2 EE3 EE4 EE5 EE6 EE7 EE8
EE9 EE10 EE11 EE12 EF1 EF2 EF3 EF4 EF5 EF6
EF7 EF8 EF9 EF10 EF11 EF12 EG1 EG2 EG3 EG4
EG5 EG6 EG7 EG8 EG9 EG10 EG11 EG12 EH1 EH2
EH3 EH4 EH5 EH6 EH7 EH8 EH9 EH10 EH11 EH12
